# Eagle Syndrome as a Delayed Vascular Complication: Carotid Stent Deformation After Stenting for High Cervical Stenosis

**DOI:** 10.7759/cureus.85205

**Published:** 2025-06-01

**Authors:** Takahiro Kumono, Tatsuya Tanaka, Tomoyuki Naito, Akira Matsuno

**Affiliations:** 1 Department of Neurosurgery, International University of Health and Welfare, Narita Hospital, Narita, JPN

**Keywords:** carotid artery stenting, eagle syndrome, elongated styloid process, elongated styloid syndrome, in-stent restenosis, internal carotid artery stenosis, neuroendovascular therapy, percutaneous transluminal balloon angioplasty

## Abstract

Eagle syndrome, caused by elongation of the styloid process or calcification of the stylohyoid ligament, can present with a variety of symptoms. The vascular variant, stylocarotid syndrome, involves mechanical compression of the internal carotid artery (ICA) and may lead to cerebrovascular complications such as dissection, transient ischemic attacks, or ischemic stroke. While carotid artery stenting (CAS) is widely used for carotid stenosis, mechanical complications related to surrounding bony structures remain rare.

We report a case of a 75-year-old woman who underwent CAS for high cervical ICA stenosis. Although the procedure was initially successful, the patient later developed recurrent ischemic stroke. Imaging revealed in-stent restenosis caused by deformation of the carotid stent, which was in close proximity to an elongated styloid process (44 mm). The patient underwent percutaneous transluminal angioplasty, followed by transcervical styloidectomy. After the final intervention, the patient remained free of restenosis or stroke recurrence during a one-year follow-up period.

This case highlights the importance of evaluating bony anatomical structures in patients undergoing CAS, particularly for high cervical lesions. An elongated styloid process may cause delayed mechanical stent deformation and restenosis. Early recognition and consideration of styloidectomy may help prevent complications and improve long-term outcomes in vascular Eagle syndrome.

## Introduction

Eagle syndrome was first described by Watt W. Eagle in 1937 and is characterized by abnormal elongation of the styloid process or calcification of the stylohyoid ligament [1]. Elongation of the styloid process, typically defined as greater than 25 mm, can compress adjacent neurovascular structures, potentially leading to a variety of symptoms [2]. Common manifestations include pharyngeal pain, otalgia, dysphagia, and cervical discomfort, and the condition is most often diagnosed in the field of otolaryngology through physical examination or imaging studies [2].

The vascular variant of Eagle syndrome, known as stylocarotid syndrome, results from compression of the internal carotid artery (ICA) by an elongated styloid process. This condition can lead to serious cerebrovascular complications, including carotid artery dissection, transient ischemic attack (TIA) [3,4], and ischemic stroke, necessitating accurate diagnosis and timely intervention [3,4].

In this report, we present a rare case of in-stent restenosis due to mechanical deformation of a carotid artery stent caused by an elongated styloid process following carotid artery stenting (CAS) for high cervical carotid stenosis. We also review the relevant literature to underscore the clinical importance of evaluating bony anatomical relationships in patients undergoing CAS.

## Case presentation

**Figure 1:**
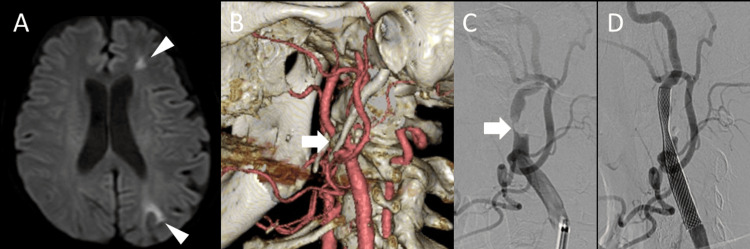
Imaging findings before and after the initial carotid artery stenting. (A) Diffusion-weighted MRI showing cerebral infarction in the left watershed area (arrowheads). (B) Three-dimensional CT angiography (3D-CTA) revealing carotid artery stenosis with surrounding calcification at the C2 vertebral level (arrow). (C) Digital subtraction angiography (DSA) of the left common carotid artery demonstrating severe stenosis (95% by the North American Symptomatic Carotid Endarterectomy Trial (NASCET) criteria) of the left internal carotid artery (arrow). (D) Post-stenting DSA showing improvement of stenosis to 39% by the NASCET criteria.

A 75-year-old woman presented to our hospital with right upper limb weakness. Her medical history included hypertension, diabetes mellitus, and cerebral infarction. Brain MRI at admission revealed an acute cerebral infarction in the left cerebral watershed area (Figure 1A).

Magnetic resonance angiography (MRA) demonstrated stenosis of the left cervical ICA, and carotid ultrasonography revealed severe stenosis with a 77% stenosis rate according to the North American Symptomatic Carotid Endarterectomy Trial (NASCET) criteria, and a peak systolic velocity (PSV) of 337 cm/s. Additionally, three-dimensional computed tomography angiography (3D-CTA) of the neck showed ICA stenosis with calcification at the level of the C2 vertebra (Figure 1B). The patient was diagnosed with atherothrombotic cerebral infarction and started on dual antiplatelet therapy consisting of aspirin (100 mg/day) and cilostazol (200 mg/day).

Approximately six months after the onset of cerebral infarction, carotid artery stenting (CAS) was performed to prevent recurrence (Figures 1C, 1D). CAS was selected instead of carotid endarterectomy (CEA) due to the patient’s advanced age, the high cervical location of the stenotic lesion, and reduced cardiac function, which collectively posed increased surgical risks. Under local anesthesia, an 8-Fr long sheath was inserted via the right femoral artery, and an 8-Fr OPTIMO guiding catheter (Tokai Medical Products, Kasugai, Japan) was advanced to the left common carotid artery. Intravenous heparin was administered to maintain an activated clotting time (ACT) of 250 seconds. A distal protection device (FilterWire EZ, Boston Scientific, Marlborough, USA) was deployed in the ICA. After inflating the balloon of the guiding catheter, the device was advanced across the stenotic lesion. Predilation was performed using a 2.5 × 40 mm SHIDEN balloon catheter (Kaneka Medix, Kita-Ku, Japan) at 8 atm for 30 seconds. A Carotid Wallstent Monorail (8 × 29 mm, Boston Scientific) was deployed from the ICA to the common carotid artery. Post-dilation was performed using a 4.5 × 30 mm SHIDEN balloon catheter at 8 atm for 10 seconds. Angiography confirmed improvement in left ICA stenosis from 95% to 39% (Figures 1C, 1D). Postoperatively, the patient developed prolonged hypotension, which was managed with continuous dopamine hydrochloride infusion. No stroke occurred, and she was discharged on postoperative day seven.

**Figure 2:**
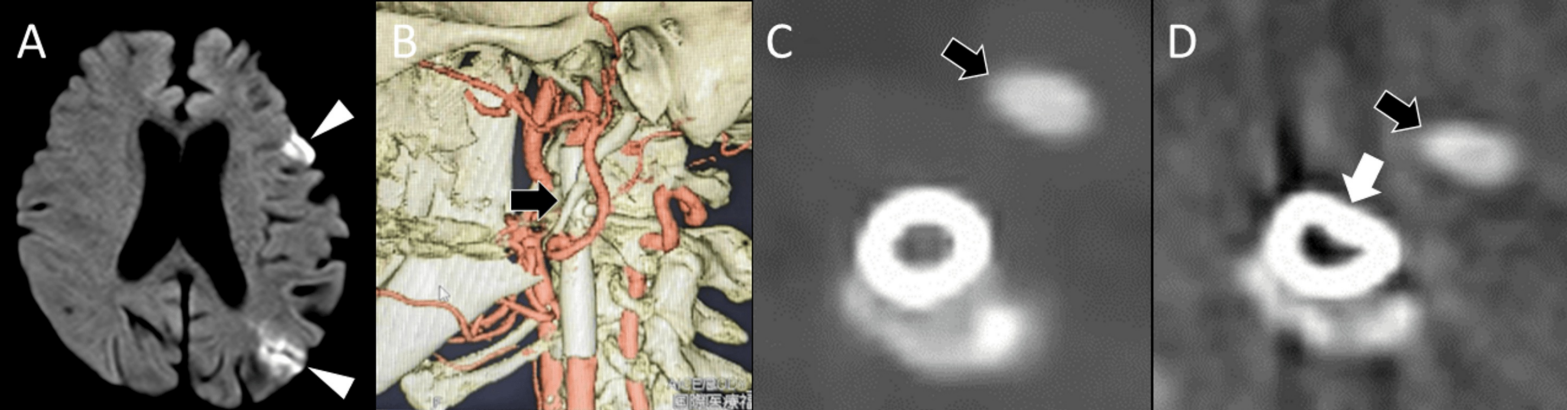
Imaging at the time of stroke recurrence. (A) Diffusion-weighted MRI showing recurrent cerebral infarction in the left watershed area (arrowheads). (B) Three-dimensional CT angiography (3D-CTA) revealing a close proximity between the carotid stent and the elongated styloid process (black arrow). (C) Post-carotid artery stenting CT showing no stent deformation immediately after the procedure. (D) At the time of stroke recurrence, the stent adjacent to the styloid process (black arrow) was deformed (white arrow).

Approximately one year and eight months after the initial stroke, the patient developed sudden-onset dysarthria and was emergently transported to our hospital. Brain MRI revealed a new acute infarction in the left watershed area (Figure 2A).

3D-CTA and plain CT scan of the neck demonstrated deformation of the carotid stent (Figures 2B-2D). Notably, an elongated styloid process (44 mm) was found in close proximity to the deformed segment (Figure 2B), suggesting mechanical compression as the cause of restenosis and hemodynamic cerebral infarction. Carotid ultrasound showed partial stent underexpansion in the left ICA. Although distal flow signals were present, no evidence of intimal hyperplasia or accelerated flow was observed. The PSV was 47.8 cm/s. The patient was started on dual antiplatelet therapy with aspirin (100 mg/day) and clopidogrel (75 mg/day) and was discharged.

At approximately one year and 11 months post infarction, follow-up carotid ultrasonography again demonstrated stent underexpansion in the left ICA. The distal segment of the stent was poorly visualized and showed marked flow acceleration, with a PSV of 226.2 cm/s. Progressive stent deformation was evident, and additional treatment was deemed necessary. Percutaneous transluminal angioplasty (PTA) was planned, followed by styloidectomy.

**Figure 3:**
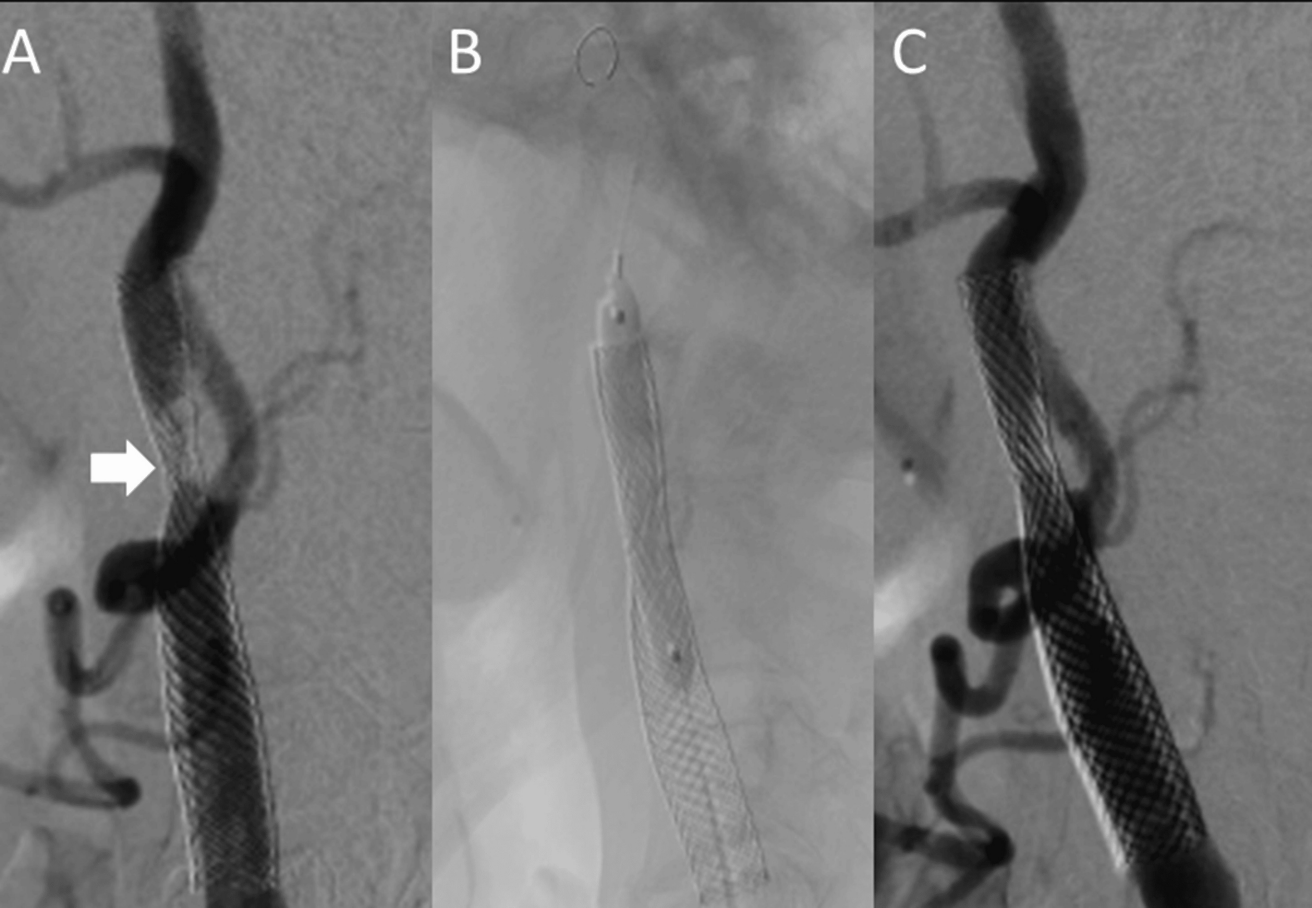
Percutaneous transluminal angioplasty (PTA). (A) Digital subtraction angiography (DSA) of the left common carotid artery showing in-stent restenosis at the site of stent deformation (arrow). (B) Balloon angioplasty being performed at the stenotic segment. (C) Post-procedure imaging showing improved patency of the internal carotid artery.

At two years and five months post infarction, PTA was performed to address in-stent restenosis of the left ICA (Figure 3).

An 8.2-Fr long sheath was inserted via the right femoral artery, and an 8-Fr OPTIMO guiding catheter (Tokai Medical Products) was advanced to the left common carotid artery. A Filter Wire EZ (Boston Scientific) was deployed at the C2 level to prevent distal embolization. A SHIDEN 4.5 × 40 mm balloon catheter (Kaneka Medix) was advanced to the stenotic site; however, due to balloon rupture, it was replaced with a 4.0 × 40 mm balloon, which was inflated at 8 atm for 30 seconds. Improvement in stenosis was confirmed, and postoperative ultrasonography demonstrated improved blood flow velocity.

**Figure 4:**
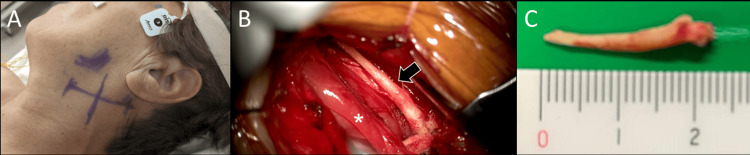
Intraoperative findings of styloidectomy. (A) A 5-cm transverse skin incision was made in the left cervical region. (B) The elongated styloid process (black arrow) was identified cranial to the posterior belly of the digastric muscle (*). (C) The resected elongated styloid process.

At two years and eight months after the initial stroke, the patient underwent styloidectomy to treat the elongated styloid process (Figure 4).

A transcervical approach was used, with a 5-cm transverse incision made one fingerbreadth below the mandibular angle (Figure 4A). The posterior belly of the digastric muscle was retracted caudally to expose and resect the styloid process (Figures 4B, 4C). Postoperative CT confirmed complete resection of the styloid process adjacent to the ICA.

Postoperative CT revealed recurrent stent deformation, indicating that compression had recurred between the PTA and styloidectomy. Therefore, a stent-in-stent procedure was scheduled for February 8, 2024. An 8.2-Fr long sheath was inserted via the right femoral artery, and an 8-Fr OPTIMO guiding catheter was advanced to the left common carotid artery. A Filter Wire EZ was placed at the C2 level. Predilation was performed using a SHIDEN 5.0 × 30 mm balloon catheter at 14 atm for two minutes. A Carotid Wallstent (8.0 × 22 mm, Boston Scientific) was then deployed. After confirming adequate stent expansion with post-dilation, the filter was removed, and the procedure was completed.

No postoperative complications occurred, and during one year of outpatient follow-up, there was no recurrence of cerebral infarction or evidence of restenosis in the left ICA.

## Discussion

Elongation of the styloid process, also known as Eagle syndrome, is a condition in which the styloid process or its associated ligament becomes abnormally elongated, leading to a variety of symptoms [2]. Among vascular complications, ICA dissection is the most frequently reported manifestation [3]. As for treatment, CAS followed by styloidectomy has been recommended in some cases [4]. However, a temporary discontinuation of antiplatelet therapy is often necessary before and after styloidectomy, which may make it difficult to perform the resection promptly following stent placement [4]. The relationship between styloid process elongation and carotid artery stenosis is clinically important because anatomical proximity may result in direct contact, potentially causing arterial dissection or thrombus formation [5]. In particular, when stenosis involves a high cervical segment, repetitive microtrauma from the elongated styloid process may contribute to vascular injury and result in dissection [6]. In the present case, the lesion was located at a high cervical level, suggesting that microtrauma from the styloid process may have played a role in the pathogenesis. However, since the styloid process was not in direct contact with the ICA and no dissection was observed, the condition was initially considered indistinguishable from atherosclerotic carotid stenosis. Nevertheless, since the deformed portion of the stent was located adjacent to the styloid process, the elongation was deemed the most likely cause of the stenosis.

In cases where the styloid process lies close to the carotid artery, the possibility of mechanical deformation of the stent should be considered, and surgical resection of the styloid process may be indicated [7,8]. There have also been reports of carotid stent damage associated with an elongated styloid process, including deformation of the CASPER stent and fracture of flow diverters [7,8]. When performing CAS in patients with this anatomical abnormality, there is a risk of stent deformation due to mechanical compression, and this risk should be carefully considered during treatment planning. In the present case, the elongated styloid process was adjacent to the ICA at the time of initial CAS but was not in direct contact, and therefore, styloidectomy was not performed. When carotid stenosis is suspected to be related to Eagle syndrome, the preferred treatment is CEA combined with styloidectomy [9]. If CEA is not feasible, CAS followed by delayed styloidectomy may be considered as a second-line approach [10,11]. During the interim period prior to styloidectomy, careful radiologic follow-up is essential to monitor for progressive stent deformation. If such deformation occurs, PTA may be used as a temporary measure.

In summary, an elongated styloid process is closely associated with carotid artery pathology and may lead to complications such as arterial dissection, stenosis, or stent deformation. A careful, individualized treatment approach is essential, along with appropriate long-term follow-up to prevent recurrence and optimize clinical outcomes. This report describes a single case, and thus, generalizability is limited. Additionally, histopathological analysis of the deformed stent was not performed, and the mechanical mechanism remains speculative.

## Conclusions

This case highlights the importance of recognizing an elongated styloid process as a potential cause of carotid artery stenosis and stent deformation. In cases of severe carotid stenosis, preoperative anatomical assessment is essential. When close proximity between the styloid process and the carotid stent is observed, early consideration of styloidectomy may help prevent mechanical complications and improve long-term outcomes.
